# Epithelial-Mesenchymal Transition in Pancreatic Cancer: A Review

**DOI:** 10.1155/2017/2646148

**Published:** 2017-12-12

**Authors:** Shuai Wang, Shuai Huang, Yu Ling Sun

**Affiliations:** Institute of Hepatobiliary and Pancreatic Diseases, School of Medicine, Department of Hepatobiliary and Pancreatic Surgery, The First Affiliated Hospital of Zhengzhou University, Zhengzhou University, Zhengzhou, China

## Abstract

Pancreatic ductal adenocarcinoma (PDAC) is one of the most aggressive solid malignancies and is characterized by its insensitivity to current therapy. The invasion and metastasis of solid tumors such as PDAC are complex processes involving many factors. Recent insights into the role of cancer stem cells (CSCs) and the epithelial-mesenchymal transition (EMT) in tumorigenesis have increased the knowledge base and highlighted new therapeutic targets of this disease. The process of EMT is regulated by a complex network of cytokines, transcription factors, growth factors, signaling pathways, and the tumor microenvironment, exhibiting CSC-like properties. The transition of solid cancer cells from an epithelial to a mesenchymal phenotype increases their migratory and invasive properties, thus promoting metastasis. In PDAC, the exact influence of EMT on the biological behaviors of cancer cells and its impact on clinical therapy remain controversial, but the therapeutic strategy of combining EMT inhibition with chemotherapy deserves attention. Alternatively, anti-inflammatory therapy that targets the interaction between inflammation and EMT is a valid strategy for treating the premalignant stage of tumor progression. In this review, we summarize the latest research on EMT and the potential relationship between EMT and PDAC.

## 1. Introduction

Pancreatic ductal adenocarcinoma (PDAC), a serious global health burden, is the eighth most common cause of cancer-related death worldwide. PDAC has an extremely poor prognosis, with a 5-year overall survival rate of less than 5.0% [1, 2]. In China, the median survival time of PDAC patients is approximately 7.8 months. Among PDAC patients, 30.0% receive curative radical operations, whereas only 9.8% undergo comprehensive effective treatment [3] due to the controversy regarding the fundamental causes of the disease. The low survival rate of PDAC can be attributed to an aggressive biological phenotype that is characterized by early local invasion and metastasis [2, 4, 5]. Accordingly, there is an urgent need to elucidate the molecular mechanisms associated with the occurrence, development, therapeutic resistance, and metastasis of this lethal disease. Numerous studies have demonstrated that the invasiveness of pancreatic cancer correlates with the epithelial-mesenchymal transition (EMT) [6–9].

EMT is a morphologic cellular program simply defined as the phenotypic transition from an epithelial to a mesenchymal state. In vivo, intermediate hybrid epithelial and mesenchymal phenotypes are frequently observed and are referred to as partial EMT. The mesenchymal phenotype is considered “metastable,” whereas the epithelial phenotype is thought to be stable and capable of colonization. EMT is modulated by complex regulatory networks involving epigenetic modification and transcriptional control, including EMT-inducing transcription factors (EMT-TFs) such as SNAIL (Zinc finger protein SNAIL), ZEB (Zinc finger E-box-binding homeobox 1), and Twist (Twist Basic Helix-Loop-Helix Transcription Factor 1) and transcription regulators, such as microRNAs (miRNAs). Regarding metastasis, numerous studies have shown that most circulating tumor cells express both epithelial and mesenchymal markers, emphasizing the crucial role of EMT during carcinoma dissemination [10–12]. Furthermore, studies have demonstrated that loss of SNAIL or ZEB, which are strong epithelial EMT repressors, in pancreatic cells does not prevent PDAC metastasis, thus opposing the classical notion of EMT [13]. EMT occurs at the tumor invasive front, which is functionally different from the bulk of tumor [14, 15] and shows cancer stem cell (CSC) involvement [16]. Moreover, correlations between some EMT-TFs associated with pancreatic carcinoma and stemness have been verified because TFs such as ZEB1, a stemness repressor that inhibits miRNA, often promote stemness. Similar to metastasis and CSCs, EMT exhibits a significant association with resistance to chemotherapy.

In this review, we discuss the roles of EMT in the dissemination and metastasis of pancreatic cancer, explore the relevance of CSCs in the tumorigenesis, colonization, and metastatic processes, and discuss the role of the inflammation environment in promoting EMT in premalignant lesions. We also discuss potential therapies and patient selection criteria based on chemotherapy according to the association between EMT and chemoresistance with the aim of improving the prognosis of patients with pancreatic cancer.

## 2. EMT Regulation

**Figure 1:**
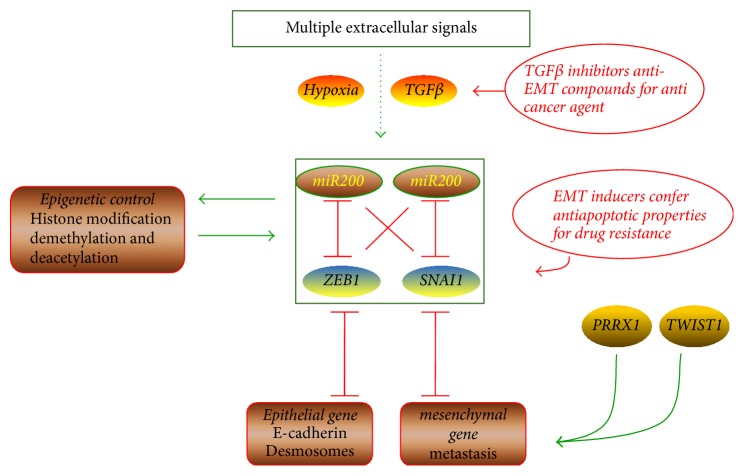
Regulatory network for EMT: key sites conferring chemoresistance as well as anti-EMT agents for cancer therapy.

EMT is regulated by a complex network involving epigenetic modifications, transcriptional control, alternative splicing, protein stability, and subcellular localization [17–19]. Some pathways might be crucial for a given EMT event during tumor progression such as differentiation, metastasis, and tumorigenesis. Members of the transforming growth factor-*β* (TGF-*β*) superfamily have been implicated as major EMT induction signals during almost all morphogenetic events, including tumorigenesis and metastasis [20]. EMT-TFs, such as SNAIL, ZEB, and Twist, are considered master regulators of these complex networks (Figure 1). In addition, multiple miRNAs, including miR-200 and miR-34, establish a negative feedback loop to maintain epithelial and mesenchymal homeostasis [17, 21, 22].

TGF-*β* signaling is considered a primary EMT inducer, although the precise pathways activated by individual family members may differ during different morphologic events, some of which include direct phosphorylation by ligand-activated receptors of SMADs [SMA (small body size) and MAD (mothers against decapentaplegic) related proteins] TFs and by certain cytoplasmic proteins regulating cell polarity and tight-junction formation [23]. TGF-*β* also influences multiple other EMT-inducing signal transduction pathways, involving Notch, Wnt (wingless (wg)/int (proto-oncogene integration-1) related genes), and integrin signaling pathways, which act in concert to trigger EMT [24].

The EMT-TFs SNAIL and ZEB are defined as potent epithelial repressors rather than mesenchymal promoters, whereas PRRX and Twist are considered strong mesenchymal inducers. SNAIL and ZEB repress the expression of epithelial markers, for example, E-cadherin, claudins, and occludins, which are downstream of this regulatory loop. Due to the “metastable” mesenchymal phenotype, the aforementioned EMT-TFs are important metastasis promoters, especially ZEB [25]. The miR-200 family is incongruously associated with both reduced invasion [26, 27] and increased metastasis [28]—which resulted in combined suppression of the EMT-promoting transcription factors SNAIL1/2, Twist, ZEB1, and ZEB2 but had no effect on metastasis. In turn, ZEB1 represses expression of the miR-200 family, including the stemness-inhibiting miRNA miR-203. This negative feedback highlights the importance of EMT-TFs in stemness and the relevance of EMT regulation and stemness maintenance to pancreatic tumors. In addition, PRRX1 (Paired Related Homeobox 1), an EMT inducer in the SNAIL1-independent pathway, drives invasiveness together with TWIST1 [29].

EMT can be regulated by upstream and downstream epigenetic changes, largely affecting the EMT miRNA-SNAIL-ZEB networks, as these EMT transcriptional drivers are involved in a complex epigenetic regulatory loop [30]. The major mechanisms of epigenetic regulation consist of histone modifications, DNA methylation, and X-chromosome inactivation [31, 32]. Simultaneous with EMT progression, cells in the tumor invasive front differentiate into an invasive subtype, and the epigenome of these cells is continually undergoing demethylation and deacetylation. In this process, SNAI1 activates chromatin modifiers such as lysine-specific demethylase 1 (LSD1) [33], G9a (euchromatic histone lysine methyltransferase 2) and Suv39H1 (suppressor of variegation 3-9 homolog 1) histone methyltransferases [34, 35], SIN3A (SIN3 transcription regulator family member A), histone deacetylase 1 (HDAC1), and HDAC2 [36], and ZEB1 participates by recruiting the LSD1-containing corepressor complex, HDAC1 and HDAC2 [37]. miR-200 family expression is also mediated by histone demethylase and DNA methylation [38]. Expression of different TF combinations caused by altered histone acetylation marks an essential relevant phenotypic change during EMT [39, 40], and EMT-TFs contribute to pluripotency by controlling chromatin and nuclear organization to modulate the transcription of downstream EMT genes.

Further research into the regulation network might provide insight into promising treatments for fatal tumors, especially pancreatic cancer.

## 3. Roles of EMT in Pancreatic Cancer Metastasis

**Figure 2:**
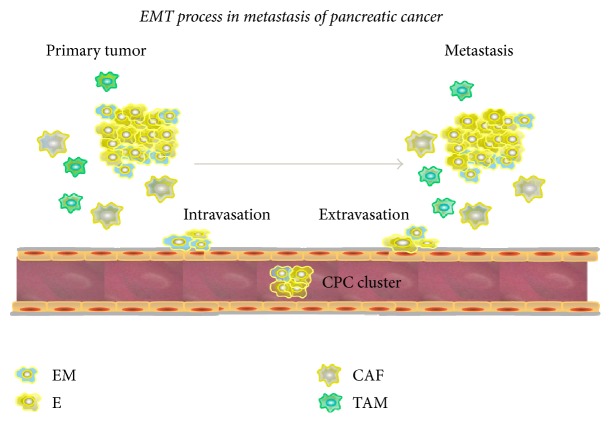
EMT during the metastatic process of pancreatic cancer. Circulating tumor cells (CTCs) or circulating pancreatic cells (CPCs) derived from primary tumor cells in pancreatic cancer undergo EMT and transition to hybrid phenotypes at the invasive front. CPC clusters colonize a distant site via the circulation, where, according to some studies, the cluster undergoes the reverse of EMT. EM: the hybrid phenotype; E: epithelial phenotype; CAF: cancer-associated fibroblast; TAM: tumor-associated macrophage.

The role of EMT in metastasis is a longstanding source of controversy, largely due to an inability to monitor transient and reversible EMT phenotypes in vivo. The hybrid phenotype, termed partial EMT, has the potential to promote or reverse EMT and is referred to as “metastable” [41]. Metastasis consists of dissemination and colonization, and, via EMT, circulating tumor cells play a vital role in this process. As numerous data have indicated, the proportion of carcinoma cells undergoing EMT in primary tumors is low [42, 43]; however, most CTCs exhibit both epithelial and mesenchymal traits, indicating that EMT might occur during dissemination of carcinoma cells, including pancreatic cancer cells [10–12]. In the presence of high concentrations of TGF-*β*-associated platelets, CTCs might originate from carcinoma cells that undergo EMT in the primary tumor or from intermediate EMT phenotypes in circulation. In addition, CTCs display a full range of EMT phenotypes, indicating that these cells express ECM components. The relative enrichment of mesenchymal markers in these cells is indicative of their successful colonization of distant organs, confirming increased abilities to invade and intravasate, although this occurs rarely in pancreatic cancer [44]. At a distant site, CTCs transitioning to the mesenchymal phenotype comprise distinct clonal carcinoma cells held together through intercellular adhesion, emphasizing the requirement for mesenchymal cancer cells to revert to the epithelial state for metastatic growth (Figure 2).

Nevertheless, untransformed epithelial cancer cells can reach distant organs, such as the lung, after traveling to the bloodstream via extravasation. Because epithelial cells, including cell clusters, can efficiently disseminate and extravasate, the EMT/MET pathway might be indispensable in all cells for secondary neoplasm generation [45]. In breast cancer, a mesenchymal-specific Cre-mediated fluorescent marker switch system in spontaneous breast-to-lung metastasis models is used to monitor transient and reversible EMT phenotypes in vivo. The research confirms that inhibiting EMT by overexpressing miR-200 did not impact lung metastasis development [42]. Moreover, studies have investigated the role of EMT in PDAC by generating mouse PDAC models with deletions of SNAIL or Twist, two key EMT transcription factors suppressed by miR-200 family, and the results showed that EMT suppression does not block the development of invasive PDAC, systemic dissemination or metastasis [13]. To some degree, these results contradict the necessity for the original EMT/MET hypothesis during tumor progression [41, 46, 47]. Based on cooperation between multiple EMT-TFs, it is possible that the loss of individual factors may not prevent cell invasion and dissemination, particularly in pancreatic cancer, in which even normal cells without strong epithelial properties are present [48]. Furthermore, there is evidence that cells in the primary tumor undergo EMT [49].

Intriguingly, invasiveness and dissemination to the bloodstream occur very early during EMT, even before a malignant tumor can be detected. Cells from pancreatic intraepithelial neoplasia (PanIN) lesions, the premalignant lesions that undergo EMT, exhibit invasive traits in vitro [6]. Lineage tracing has suggested that pancreatic cells can delaminate from PanIN lesions and cross the basement membrane, which encases circulating pancreatic cells (CPCs), before such invasive behavior is detected and lesions have seeded to distant organs. CPCs are derived from acinar cells undergoing EMT, and these cells acquire survival and self-renewal properties called stem cell-like characteristics that could be considered tumor-initiating properties. These results are consistent with the finding that because of metastatic disease, the mortality of patients who receive surgical resection of small pancreatic neoplasms with clear surgical margins and no evidence of metastasis is over 75% within 5 years [50]. Additionally, a fraction of patients who receive a pancreatectomy for chronic pancreatitis with PanIN develop metastatic PDAC. Furthermore, inflammation facilitates EMT, and an increase in the number of CPCs was observed in Pdx1-Cre; Rosa^YFP^ and Pdx1-Cre; Kras^G12D^; Rosa^YFP^ mouse pancreatitis models [6], to some degree, which could prove the significant role inflammation plays in the PDAC metastasis.

Indeed, EMT might facilitate invasion and intravasation of other cells that retain their epithelial characteristics, and it plays a crucial role in early metastasis, even at the premalignant lesion stage. Nonetheless, some authors have questioned whether EMT is a prerequisite for metastasis, especially for colonization [13].

## 4. Cancer Stem Cells Undergo EMT in Pancreatic Cancer

Considering the above data, cells undergoing EMT appear to acquire stem-like features, indicating the crucial role of EMT in CSC generation. CSCs, a small subgroup of tumorigenic cells within tumors, can self-renew, differentiate, survive under stress, and metastasize. CSCs are often identified using a number of cell surface markers, including CD44, CD24, and CD133, which are essential for detecting CSCs in a cluster undergoing EMT [51, 52]. CSCs, which express traits of both stem cells and cancer cells, have been identified in tumors [53], and based on cell division symmetry and gene expression alterations, CSCs differ from other cells within the tumor [54]. These findings emphasize the crucial role of EMT in cancer recurrence and metastasis.

EMT-TFs, including members of the SNAIL and ZEB families, play crucial roles in the gland-reconstituting activity of stem cells as master regulators [55]. ZEB1 is described as an EMT inducer and transcriptional repressor that in particular represses stemness-inhibiting miR-203 and miR-200 family members. Downstream miR-200 family members, including Sox2 (sex determining region Y-Box 2) and Klf4 (Kruppel-like factor 1), are also stem cell factors. Additionally, the correlation between ZEB1 and stemness is measured by the extent of sphere formation in undifferentiated pancreatic cancer cells [25]. For example, ZEB1 knockdown results in poor initiation of sphere formation and a further reduction in sphere number after in vitro migration, indicating that ZEB1 is necessary for self-renewal. Pancreatic CSCs are defined as a CD24+/CD44+ subpopulation [56], and decreased CD24 expression in ZEB1-knockdown cells has been confirmed. However, Snail1 mediates a shift from asymmetric (one stem cell, one differentiating cell) to symmetric (two stem cells) cell divisions, showing that EMT has a role increasing CSC numbers in the tumor stem cell reservoir [57]. TGF-*β* is defined as the main and crucial EMT inducer during cancer pathogenesis progression [23] and dramatically enhances the program by which cancer-associated fibroblasts (CAFs) increase the frequency of tumor-initiating cells in cancer patients [53].

Nevertheless, there are numerous data for the uncoupling of EMT and CSCs. According to some evidence, EMT and CSCs can be defined as in parallel pathways rather than in the same pathway, especially during metastatic colonization. It is widely accepted that disseminated tumor cells (DTCs) undergo MET to transition toward a more epithelial phenotype for colonization, and this process is regulated by metastasis-associated macrophages (MAMs) that suppress EMT-TFs [58]. By converting hepatic stellate cells into myofibroblasts, MAMs also play an essential role sustaining metastatic tumor growth [59]. Regarding EMT-TFs, the lack of PRRX1, an EMT promoter, induces the proliferation of clusters and results in mammosphere formation and the emergence of a CD44-high cell cluster, which is considered a stem cell subpopulation [29]. Furthermore, fibroblast reprogramming is also required for EMT during the induction of tumor-initiating capacities. Oct-4 (octamer-binding protein 4), Klf4, c-Myc (Myc proto-oncogene), and Sox2 sequentially induce a high rate of pluripotency [60], and, as mentioned previously, Klf4 and Sox2 are stem cell factors. Due to such sequential induction, epigenetic reorganization is thought to occur during the interval between stemness and EMT [61].

Intriguingly, the upregulation of SNAIL1 expression in prostate and bladder cancer cells suppresses stemness, which contrasts with other relevant data associating EMT and stemness [62] and resembles the lethal EMT process induced by TGF-*β* in PDAC [63]. In TGF-*β*-sensitive PDAC cells, EMT becomes lethal by changing TGF-*β*-induced Sox4 from a tumorigenic inducer to an apoptosis promoter, evidence that supports the uncoupling of EMT and stemness, especially in PDAC.

However, an intermediate EMT state might help to enrich for cells that are prone to exhibiting CSC-like traits, which has been observed in pancreatic cells. The intermediate phenotype derived from epithelial cells undergoing partial EMT displays both epithelial and mesenchymal traits, providing an essential condition for dissemination and colonization [11].

MAP3K4/CBP (mitogen-activated protein kinase 4) activity represses epithelial gene expression, causing TS cells to transition to an intermediate EMT state while maintaining their stem cell properties [15, 39], similar to observations in pancreatic cancer cells. Transplantation of YFP^+^E-cad^+^ or YFP^+^E-cad^−^ pancreatic cells from PDAC mice into pancreata results in histologically similar tumor formation. Additionally, all of these cells expressed either ZEB1 or E-cad at comparable levels in both groups, indicating EMT progression. Thus, the E-cad status appears to be important for tumorigenesis or stemness. In other words, there is significant plasticity between the epithelial and mesenchymal states considered to be partial EMT [6].

## 5. Inflammation Promotes EMT in PanIN

As mentioned previously, EMT-TF-miRNA networks, for example, the CSC/ZEB1–miR-200 feedback loop, not only explain the mechanism of EMT but also demonstrate its correlation with cancer progression. Because TGF-*β* plays a role in inflammation, the TGF-*β* pathway and resulting inflammation have been implicated in EMT.

Inflammation plays decisive roles at different stages of tumor development, including initiation, promotion, malignant conversion, invasion, and metastasis [64]. In the primary tumor boundary, the persistent recruitment of immune cells, such as macrophages and lymphocytes, is considered a necessary event for establishing the inflammatory tumor microenvironment. Additionally, remarkable interactions between tumor-associated macrophages (TAMs) during tumor cell dissemination and invasion indicate a correlation with EMT [65, 66]. In pancreatic cancer, inflammation is defined as a significant event during carcinogenesis and premalignant lesion progression [67, 68]. Additionally, a strong correlation between chronic pancreatitis and pancreatic cancer is now widely accepted [69].

By increasing the number of circulating cancer cells via EMT, inflammation facilitates invasion and dissemination. Additionally, CPCs are known to play a critical role during the metastatic progression of pancreatic cancer and premalignant lesions. CPCs are derived from acinar cells undergoing EMT, which are considered the initial tumor cells; thus, CPCs in circulation might indicate EMT at the primary tumor site. Recently, acute pancreatitis was induced using cerulean and pancreatic duct ligation in KCY mice. As expected, this treatment resulted in the formation of acinar-to-ductal metaplasia with inflammation (ADMIs), PanIN, and defects in epithelial characteristics. Moreover, compared with the source pancreas and sham-treated group, numerous CD24^+^CD44^+^ CPCs (defined as possessing stemness qualities) were detected and strongly increased in the circulation, which is consistent with the observations from the natural PanIN condition. For further verification, posttreatment with dexamethasone resulted in almost complete elimination of PanIN lesions in the pancreas and CPCs in the circulation, highlighting a crucial prerequisite for maintaining PanIN lesions. These data demonstrate that expression of oncogenic Kras facilitates EMT and dissemination in response to acute pancreatitis.

At the molecular level, SNAIL is an essential EMT-TF that is stabilized in the EMT process by the inflammatory cytokine TNF-*α* (tumor necrosis factor alpha) via activation of the NF-kB (nuclear factor kappa B) pathway [70]. TAMs are essential inflammatory cells that secrete proinflammatory cytokines as TNF-*α* and facilitate angiogenesis and tissue remodeling, thus promoting tumor cell motility. Additionally, TNF-*α* activates the major inflammatory response pathway NF-kB, which facilitates both tumor development and metastatic progression [71, 72]. Because SNAIL itself is defined as a repressor of the epithelial phenotype and an inhibitor of apoptosis, the migration of cells undergoing inflammation-induced EMT increases dramatically. Furthermore, the anti-inflammatory cytokine TGF-*β* is an important regulator of EMT [23], and numerous studies have demonstrated that the TGF-*β* pathway collaborates with the Wnt, Notch, and MAPK (mitogen-activated protein kinase) pathways to facilitate EMT during various morphological processes. Additionally, the TGF*β*/Smad pathway has been shown to coordinate with Ras activation to promote EMT [73]. However, as the inflammatory microenvironment increases the likelihood of mutations, which are considered to play crucial roles in tumorigenesis, TGF-*β* suppresses epithelial cell proliferation and early tumor growth, causing some tumors to acquire inactivating mutations in TGF-*β* signaling components. Overall, the effectiveness of anti-inflammatory agents in reducing mortality bolsters the association between inflammation and tumor progression [74].

In general, if inflammation promotes EMT by stabilizing SNAIL expression and the TGF-*β* signaling pathway, then anti-inflammatory therapy is a valid strategy for treating the premalignant stage of tumor progression. Furthermore, the number of CPCs might be included as a prognostic predictor for early stage therapy.

## 6. EMT and Chemoresistance in Pancreatic Cancer

Due to a lack of early detection methods, patients are typically diagnosed at a late stage, with a five-year survival rate of <5%. Surgical resection remains the only curative treatment, but fewer than 20% of patients qualify as candidates. Chemical therapies have comprised the major available regimens in the last two decades. Unfortunately, the current chemical treatment for advanced PDAC, including gemcitabine-based combinations, molecular target therapy, and multiagent regimens, still results in a poor prognosis, with only 5.2% of patients surviving after three years [1, 2]. Although gene expression profiling has been applied to identify biomarkers and therapeutic targets in pancreatic cancer [75], the molecular mechanisms of chemoresistance in pancreatic cancer still require elucidation, and EMT might be the crucial event. Various pathways present in the cancer environment, such as chemotherapy resistance, confer resistance to death in cells undergoing EMT [76]. Although the ability of EMT to confer chemoresistance to pancreatic cancer cells is clear, cells undergoing EMT have a limited contribution to tumor progression, including during the establishment of metastases and tumorigenesis [13, 76]. Furthermore, a more “mesenchymal” phenotype exhibits higher drug resistance [77].

Immunotherapy for cancer treatment has generated substantial interest in recent years, with notable successes in several tumor types, including PDAC [2, 78, 79]. Recent studies have elucidated the essential role EMT plays in immunotherapy resistance [80–82]. Immunotherapy, which uses monoclonal antibodies against targets such as programmed death-1 (PD1) or cytotoxic T lymphocyte-associated antigen 4 (CTL4), two inhibitory T-cell receptors, has become prevalent in recent years. As expected, dominant mutations are induced by these therapies; however, these mutations do not contribute to the resistance of CTL cancer cells to lysis, and the EMT of target cancer cells actually abolishes it [83]. Controversially, cancer cells undergoing EMT might not be susceptible to CTL lysis, which has been supported by research in melanoma cells [81]. The cancer cells that undergo SNAIL-mediated EMT are considerably more metastatic than their parental cells, and this has been attributed to the emergence of T regulatory (T_reg_) CD4 cells expressing Foxp3 (Forkhead Box P3), which induces immunosuppression in these cells [81]. Despite a lack of evidence for pancreatic cancer cells, the aforementioned factor might participate in T_reg_ cell chemoresistance to drugs such as cyclophosphamide. Intriguingly, tumors that respond best to CTL-A4, and more recently to PD1 and PD1L antibodies, exhibit a higher EMT score, which indicates an increase of target cell tension potentiates killing by CTLs [84]. This research reveals an unappreciated physical dimension to lymphocyte function and demonstrates that cells use mechanical forces to control the activity of outgoing chemical signals, which has been proved in melanomas and renal, bladder, and NSCL carcinomas. There is little evidence to support this phenomenon in PDAC. Further research regarding the interaction between EMT and immunosuppression is needed. In addition to immunotherapy, vaccines are being evaluated in the adjuvant setting for pancreatic cancer; 5-Fluorouracil (5-FU)-based chemoradiation combined with a pancreatic cancer vaccine of irradiated granulocyte-macrophage colony stimulating factor (GM-CSF) is undergoing clinical trials, and a vaccine was recently developed against the mesenchymal-associated transcription factor brachyury, which is currently undergoing clinical trials for the treatment of solid tumors [85]. It might be the first vaccine platform aimed at targeting a driver of tumor EMT that has successfully reached the clinical stage.

In pancreatic cancer cells, the EMT-TF SNAIL is associated with antiapoptotic features and chemoresistance [86] against 5-fluorouracil (5-FU) and gemcitabine [87]. To verify these findings, KPC, KPC; Snail^cKO^ and KPC; Twist^cKO^ mice were treated with gemcitabine. As expected, the latter two groups showed improved histopathology, increased survival, and defective tumor progression, as detected by magnetic resonance imaging (MRI) [13]. Equilibrative nucleoside transporter (ENT1) and concentrating nucleoside transporter (Cnt3) were significantly upregulated in cancer cells compared with KPC cells, which might constitute the mechanism connecting EMT and chemoresistance in PDAC. Regardless, the crucial role of ZEB1 in pancreatic cell drug resistance has been demonstrated in numerous studies, and EMT inducers including Twist and SNAIL confer antiapoptosis properties [88, 89]. Following treatment with gemcitabine in ZEB1-knockdown cells, proliferation was strongly repressed, though the proliferative capacities of both control and ZEB1-knockdown clusters were similar without drug treatment [25]. Additionally, in assessing the correlation of E-cadherin expression, morphological changes were identified by light microscopy [77], with E-cadherin and ZEB1 expression showing inverse correlations. Loss of E-cadherin expression mediated by transcriptional suppression has been associated with a poor clinical prognosis in several types of cancers [90, 91]. Interestingly, as mentioned above, EMT is lethal in TGF-*β*-sensitive PDA cells because TGF*β*-induced Sox4 changes from an inducer of tumorigenesis to a promoter of apoptosis. Although they can also lead to chemoresistance, TGF-*β* inhibitors are the most intensively investigated anti-EMT compounds for use as anticancer agents.

## 7. Conclusion and Future Perspectives

Tremendous efforts and notable achievements have been made in the past few decades; however, pancreatic cancer patient survival has not improved [1, 2, 92]. With further research on the EMT process and its regulation, these cellular programs will be associated with disease progression and therapy, especially in cancer, and will reveal potential targets for treating pancreatic cancer. Whether EMT participates in pancreatic cancer dissemination and colonization remains under debate. The role of EMT-TF-miRNA appears distinct in pancreatic cancer compared with other carcinomas, such as breast cancer, indicating the diversity of EMT function in different cancers. Thus, some essential EMT regulation network factors must be reverified in pancreatic cancer. Furthermore, EMT might be indispensable for pancreatic cancer metastasis or dissemination. Although EMT is considered the crucial event of carcinoma invasion and dissemination, due to its complex regulatory networks, direct evidence linking colonization and EMT is lacking, and further studies are needed to reveal the true association between EMT and metastasis. Additionally, according to the results of an experimental model of pancreatic cancer and premalignant lesions, these programs might occur at an early stage of carcinoma, leading us to consider that EMT may be a crucial prognostic indicator. A quantitative EMT scoring system for highlighting the developmental lineage of each cancer type defined by both a micro- and macro-EMT gradient was recently established based on gene expression profiles, ranking the EMT state from −1.0 to +1.0 [43]. Additionally, each cancer stage shows a distinct propensity for diverse EMT states.

In pancreatic cancer, CPCs are crucial for both dissemination and stemness. CPCs derived from primary lesion cells intravasate into the circulation and acquire self-renewal and differentiation traits through EMT. Further probing for CPCs in colonization, tumorigenesis, and dissemination is thought to be an EMT breakthrough and unique to the progression of pancreatic tumors. Due to their emergence in premalignant lesions, CPCs in the circulation could be prognostic or diagnostic factors of the tumor process as well as a target of anti-EMT agents. The interactions of EMT with the inflammation microenvironment are worth further exploration.

EMT is proposed to be a suppressor of cancer cell proliferation, drug transporters, and concentrating proteins, all of which are involved in the chemoresistance of pancreatic cancer. Due to the tight association between chemoresistance and EMT, traditional chemotherapy drugs such as gemcitabine could be coadministered with an anti-EMT agent or a drug that reverses the EMT process. The emergence of personalized therapy strategies and recent advances in pharmacogenomics will provide distinct opportunities for individualized cancer treatment strategies. Indeed, the diversity of the EMT network in different individuals and different subpopulations and even at different stages of tumor development precisely demands individualized treatment strategies. The EMT pathway and transitional events can be implemented to target tumors and enhance current treatment strategies.
